# The transglutaminase type 2 and pyruvate kinase isoenzyme M2 interplay in autophagy regulation

**DOI:** 10.18632/oncotarget.6759

**Published:** 2015-12-24

**Authors:** Sara Altuntas, Federica Rossin, Claudia Marsella, Manuela D'Eletto, Laura Diaz Hidalgo, Maria Grazia Farrace, Michelangelo Campanella, Manuela Antonioli, Gian Maria Fimia, Mauro Piacentini

**Affiliations:** ^1^ Department of Biology, University of Rome “Tor Vergata”, Rome, Italy; ^2^ Department of Comparative Biomedical Sciences, The Royal Veterinary College London and UCL Consortium for Mitochondrial Research, London, UK; ^3^ National Institute for Infectious Diseases, IRCCS “Lazzaro Spallanzani”, Rome, Italy; ^4^ Department of Biological and Environmental Science and Technology (Di.S.Te.B.A.), University of Salento, Lecce, Italy

**Keywords:** autophagy, transglutaminase type 2, pyruvate kinase M2, LC3, Beclin1

## Abstract

Autophagy is a self-degradative physiological process by which the cell removes worn-out or damaged components. Constant at basal level it may become highly active in response to cellular stress. The type 2 transglutaminase (TG2), which accumulates under stressful cell conditions, plays an important role in the regulation of autophagy and cells lacking this enzyme display impaired autophagy/mitophagy and a consequent shift their metabolism to glycolysis. To further define the molecular partners of TG2 involved in these cellular processes, we analysed the TG2 interactome under normal and starved conditions discovering that TG2 interacts with various proteins belonging to different functional categories. Herein we show that TG2 interacts with pyruvate kinase M2 (PKM2), a rate limiting enzyme of glycolysis which is responsible for maintaining a glycolytic phenotype in malignant cells and displays non metabolic functions, including transcriptional co-activation and protein kinase activity. Interestingly, the ablation of PKM2 led to the decrease of intracellular TG2's transamidating activity paralleled by an increase of its tyrosine phosphorylation. Along with this, a significant decrease of ULK1 and Beclin1 was also recorded, thus suggesting a block in the upstream regulation of autophagosome formation. These data suggest that the PKM2/TG2 interplay plays an important role in the regulation of autophagy in particular under cellular stressful conditions such as those displayed by cancer cells.

## INTRODUCTION

TG2 is a unique pleiotropic enzyme belonging to the family of transglutaminase which catalyses post-translational modifications of proteins through Ca^2+^ dependent reactions including protein–protein crosslinking, incorporation of primary amines into proteins, as well as glutamine deamination [1-3]. TG2 is the most ubiquitous and widely distributed isoform in tissues and cell types. It is predominantly a cytosolic protein but is also present in mitochondria, nucleus, on the plasma membrane and in the extracellular matrix under pathological settings [4-6]. Under steady state conditions, TG2 is in a closed (compact) conformation, upon stress stimuli, its Ca^2+^ dependent activation shifts TG2 to undergo a conformational change (open) and it becomes catalytically active as a transamidating enzyme [7, 8]. In addition to protein transamidation, TG2 displays other enzymatic activities, which do not require Ca^2+^ such as GTPase, protein kinase, and protein disulfide isomerase (PDI) activity [9-11]. More recently, the PDI activity of TG2 has been shown to regulate mitochondrial function [12]. In fact, our group has provided the first evidences showing that TG2 contributes to the correct assembly of the respiratory chain complexes [13]. The absence of TG2's PDI activity leads to incorrect assembly of mitochondrial ADP/ATP transporter adenine nucleotide translocator 1 (ANT1) [14]. ANT1 is the most abundant protein in mitochondria and catalyses the exchange of mitochondrial ATP for cytosolic ADP [15]. Additionally, due to its multifunctional nature, TG2 is involved in the regulation of numerous cell functions, including cell death/survival and autophagy [16, 17] and many TG2 substrates have also been identified both *in vitro* and/or *in vivo* [18-20].

Autophagy is an essential homeostatic process that delivers cytoplasmic constituents to the lysosome. As a result, it is able to clear long-lived proteins, aggregates, organelles such as mitochondria, ER, peroxisomes and possibly bacteria and viruses [21-24]. Even though there is an ongoing basal autophagy in the cell, autophagy is rapidly activated in response to stress, e.g. nutrient deprivation, hypoxia and pressure overload to catabolize cellular substrates and generate energy [21-24]. In fact, metabolites released from lysosomes are involved in regulation of cellular homeostasis and energy production [25]. This suggests an essential role for autophagy in energy homeostasis. Defects in the autophagy machinery have been associated to the pathogenesis of many diseases including neurodegenerative, heart and liver disorders and cancer [22-23, 26-27]. Previous studies in our and other groups have proposed a role of TG2 in autophagy [28-30]. Particularly, TG2 and its transamidating activity have shown to be important for a proper autophagic degradation [28-29]. It has also been proposed that TG2 is involved in the formation and clearance of ubiquitinated protein aggregates characterizing diseases affecting both the brain and the liver [4, 31]. Additionally, a very recent study in our group has shown that TG2 ablation leads to mitophagy impairments and, in order to survive, the cells lacking this enzyme display a higher rate of aerobic glycolysis [32]. This important metabolic change suggests a role for the enzyme in the regulation of the cellular metabolism from mitochondrial respiration to aerobic glycolysis, which is typically detected in transformed cancer cells [33].

On these bases we decided to further investigate the molecular mechanisms by which TG2 is involved in these cellular processes by identifying its interactome under normal and autophagic conditions.

## RESULTS

### Characterization of TG2 interactome under normal and autophagic conditions

In order to identify the TG2's binding partners, we carried out the enzyme interactome analysis under steady state and autophagic conditions (induced by starvation for 2 hours) by utilizing TAP approach combined with HPLC and MALDI TOF/TOF mass spectrometry. TAP method involves the fusion of a TAP tag to the target protein and its expression in a host cell or organism [34]. The TAP-tagged protein, as well as its associated partners, is purified from cell extracts by two specific affinity purification/elution steps. Flag-hemagglutinin (HA)-tagged TG2 was expressed in human fibroblast 2fTGH cells (Figure S1A and S1D). 2fTGH cells transfected with untagged TG2 served as a negative control (Figure S1B). Western blot analysis for TG2 and sypro staining confirmed that TAP was performed successfully as there were no HA-Flag bands detected in control conditions (Figure S1B and S1C). During autophagy TG2 is acting as a transamidating enzyme assuming its open conformation versus the closed one present in cells under normal steady state conditions [28]; considering this, our analysis will also define the enzyme's protein partners associated to these two different 3D configurations.

Our interactome analysis has revealed that TG2 interacts with various proteins belonging to different functional categories such as chaperones, mitochondria, metabolism and cytoskeleton (Figure S2). Particularly, out of all categories, 39% and 50% are proteins related to chaperone protein family. In addition, we found a significant reduction in the number of TG2 interacting proteins in cells undergoing autophagy (Table 1). However, the interactions with chaperones are still observed. Interestingly, we have found that TG2 interacts with a well-defined group of proteins involved in the clearance of misfolded and toxic/mutated proteins. Among these, particularly interesting is a group of chaperones participating in the handling of proteins such as HSP10, HSP40, HSPA1A, HSP90, DnaJ and BAG2 (Table 1), some of which are components of chaperone mediated autophagy (CMA) and chaperone-assisted specific autophagy (CASA) [35-36]. In addition, we have identified the interaction of TG2 with some mitochondrial proteins such as the ATP synthase, ANT and Pyruvate Kinase M2 (PKM2), which is a rate limiting enzyme of glycolysis [37].

**Table 1:** The list of TG2 interacting proteins under normal and autophagic conditions

NORMAL			
Rank Protein Name	Accession No.	Total Ion Score	Total Ion C.I.%
HSPA1A protein [Homo sapiens]	gi|14424588	2748	100
Tubulin, beta 2 [Homo sapiens]	gi|12654709	1.657	100
Tubulin alpha 6 [Homo sapiens]	gi|15080013	1.444	100
tubulin alpha-1 chain - human	gi|2119266	1.420	100
tubulin beta	gi|223429	1.072	100
heat shock 10kDa protein 1-like [Homo sapiens]	gi|55962550	1.001	100
TUBA8 [Homo sapiens]	gi|47678731	966	100
dnaK-type molecular chaperone HSPA6 - human	gi|87626	813	100
heat shock protein 90-alpha - human	gi|72219	738	100
solute carrier family 25 (mitochondrial carrier\; adenine nucleotide translocator), member 6 [Homo sapiens]	gi|57162659	634	100
beta-tubulin 4Q [Homo sapiens]	gi|16974658	579	100
heat shock 70kDa protein 9B precursor [Homo sapiens]	gi|24234688	560	100
BAG2 [Homo sapiens]	gi|49065418	428	100
DnaJ protein homolog [Homo sapiens]	gi|219588	416	100
similar to Chain, Heat-Shock Cognate 70kd Protein (44kd Atpase N-Terminal Fragment) (E.C.3.6.1.3)	gi|51095055	407	100
DnaJ (Hsp40) homolog, subfamily C, member 7 [Homo sapiens]	gi|4507713	348	100
EEF1A1 protein [Homo sapiens]	gi|48735185	311	100
BiP protein [Homo sapiens]	gi|6470150	290	100
glyceraldehyde-3-phosphate dehydrogenase [Homo sapiens]	gi|31645	237	100
Similar to ATP synthase, H+ transporting, mitochondrial F1 complex, alpha subunit, isoform 1	gi|13938339	213	100
stress-induced-phosphoprotein 1 (Hsp70/Hsp90-organizing protein) [Homo sapiens]	gi|54696884	171	100
TSG101	gi|48425523	187	100
enigma protein isoform 1 [Homo sapiens]	gi|11496885	163	100
PKM2 protein [Homo sapiens]	gi|34782802	141	100
enigma - human	gi|627429	123	100
AUTOPHAGY			
Rank Protein Name	Accession No.	Total Ion Score	Total Ion C.I.%
Tubulin, beta 2 [Homo sapiens]	gi|12654709	452	100
Tubulin alpha 6 [Homo sapiens]	gi|15080013	386	100
TUBA8 [Homo sapiens]	gi|47678731	377	100
TUBB [Homo sapiens]	gi|49456871	336	100
solute carrier family 25, member 5 [Homo sapiens]	gi|4502099	308	100
tubulin beta chain - human	gi|71584	300	100
ADP.ATP translocase	gi|339723	294	100
solute carrier family 25 (mitochondrial carrier; adenine nucleotide translocator), member 4 [Homo sapiens]	gi|55749577	267	100
EEF1A1 protein [Homo sapiens]	gi|48735185	83	100

The complex of TG2 and its interacting partners was separated by HPLC and identified by mass spectrometry.

### Validation of TG2 interactome

**Figure 1:**
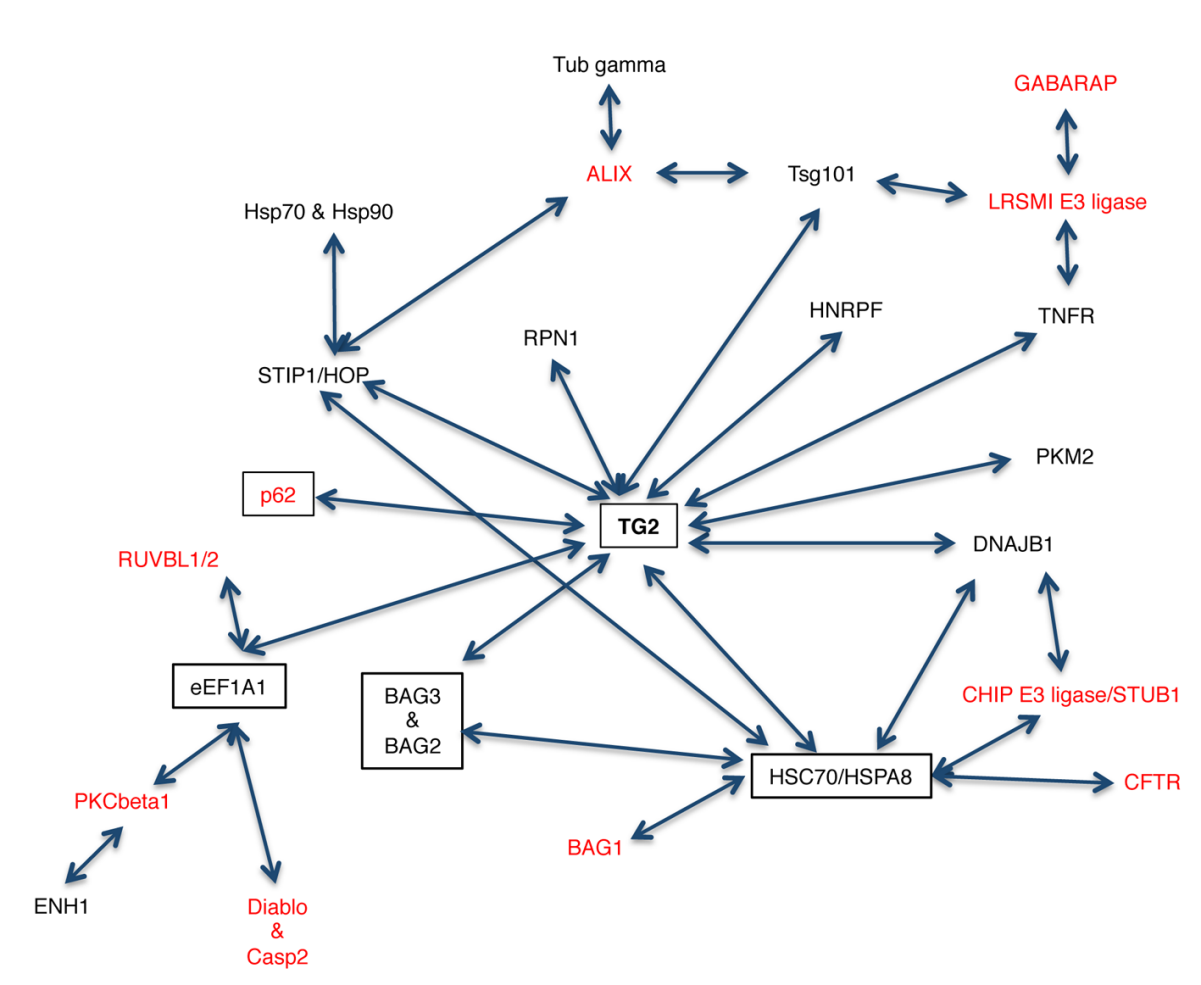
Schematic representation of the TG2 interactome The proteins in black were obtained from our TAP analysis while the ones in red were added after screening the protein/protein interaction databases. Proteins in boxes are involved in the clearance of ubiquitinated proteins.

**Figure 2:**
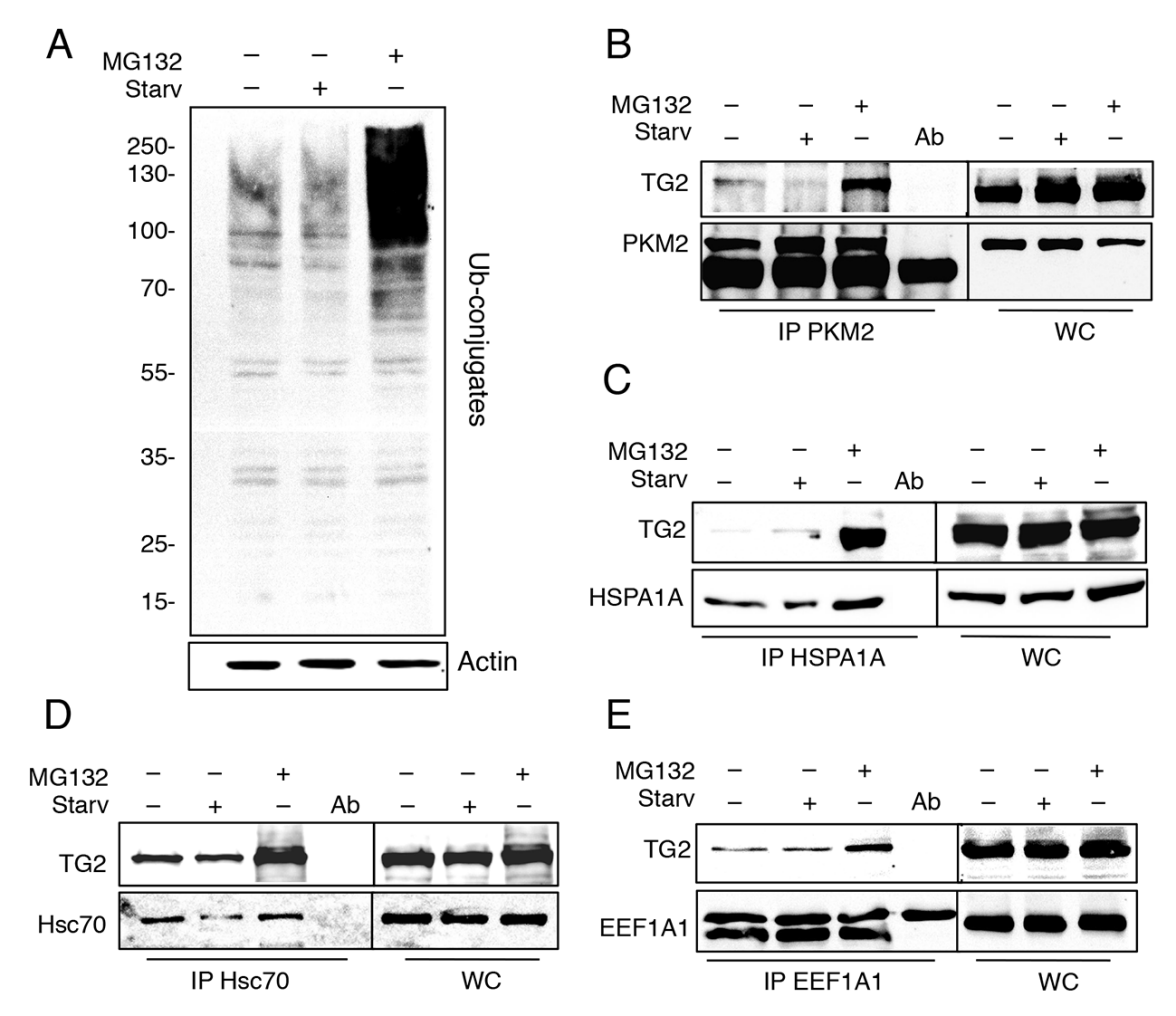
TG2 interacts with PKM2, HSPA1A, Hsc70 and EEF1A1 in Flag-HA-TG2 2fTGH cells **A**. Immunoblotting analysis of ubiquitinated proteins in 2fTGH cells. Cells were subjected to starvation in presence or absence of MG132 for 4 h, and ubiquitin expression levels were assayed by immunoblotting with anti-ubiquitin antibody. Actin was used as loading control. Immunoblotting analysis of **B**. anti-PKM2 immunoprecipitation (IP), **C**. anti-HSPA1A IP, **D**. anti-Hsc70 IP and **E**. anti-EEF1A1 IP in Flag-HA-TG2 overexpressing 2fTGH cells. Cells were subjected to immunoprecipitation, using the indicated antibodies, under steady state condition, after 4h of starvation and upon proteasome inhibition by using MG132 for 4 h. WC, whole cell lysate, was used as a protein control.

TG2 and its interacting partners identified in this study are part of a very large network (Figure 1). It is interesting to note that TG2 interacts with a network of chaperone proteins (highlighted in boxes) that are involved in the protein handling machinery, thus suggesting an important role for TG2 in proteostasis. Therefore, in order to confirm that TG2 interacts with the putative novel partners identified by the TAP approach, we analysed by co-immunoprecipitation (co-IP) the interaction of the enzyme with some proteins reported in the interactome list (Table 1) such as HSPA1A, a molecular chaperone; PKM2, involved in the glycolytic pathway; Hsc70 and EEF1A1, two important mediators of CMA [35, 38-40]. Since the interactome analysis indicated a role for TG2 in proteostasis, we decided to treat 2fTGH cells with MG132, a specific inhibitor of proteasome that is known to induce a cellular stress associated to the accumulation of misfolded ubiquitinated proteins (Figure 2A), as well as with nutrients deprivation (Starv), which leads to autophagy induction. The results confirmed that TG2 co-immunoprecipitates with all the above mentioned proteins (Figure 2B-2E). Our co-IP analysis also shows that the interaction of TG2 with these protein substrates is already detectable in basal conditions with the exception of HSPA1A, thus confirming the interactome data. On the other hand, upon starvation, TG2 interacts with HSPA1A, Hsc70 and EEF1A1, while its interaction with PKM2 is diminished upon autophagy induction (Figure 2B). Interestingly, for all the analysed proteins the stronger interaction with TG2 is detected under proteostasis stress induced blocking the proteasome by MG132.

### PKM2 regulates the transamidating activity of TG2

**Figure 3:**
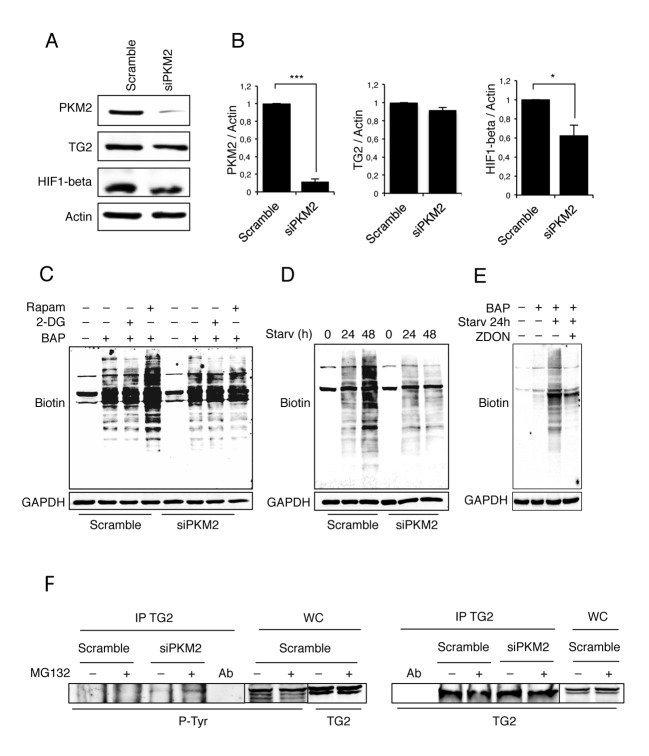
Analysis of TG2 transamidating activity and PKM2 protein expression levels **A**. Immunoblotting analysis of PKM2, TG2 and HIF1-beta in 2fTGH cells. Knockdown of PKM2 was performed by RNA interference for 72 h, as described in Material and Methods. Actin was used as a loading control. **B**. Densitometric analysis of blots (quantification of PKM2, TG2 and HIF1-beta bands normalized to actin levels). Data are expressed as mean ± SD, n = 4 independent experiments. Statistical significance refers to the respective control (*p<0.05 and ***p<0.0001). (C-D) Immunoblotting analysis of the TG-catalysed incorporation of BAP in 2fTGH cells in the presence and absence of PKM2. The cells were labelled with BAP and treated with 2-DG or Rapamycin for 4 h **C**. or subjected to starvation for 24 and 48 hours **D**. GAPDH was used as a loading control. **E**. Immunoblotting analysis of the TG-catalysed incorporation of BAP in 2fTGH cells. The cells were labelled with BAP and subjected to starvation for 24 h in absence or presence of ZDON. Cells not labelled with BAP were used as negative control. GAPDH was used as a loading control. **F**. Immunoblotting analysis of anti-TG2 immunoprecipitation in 2fTGH cells. PKM2 was knocked-down by RNA interference for 72 h as described in Materials and Methods, and cells were subjected to immunoprecipitation in steady conditions and upon proteasome inhibition with MG132 for 4 h. Anti-phosphotyrosine (p-Tyr) was used to reveal the presence of phosphorylated TG2 (left panel). Anti-TG2 was used to visualize immunoprecipitated TG2 (right panel). WC, whole cell lysate, was used as a protein control.

It has been shown that cells lacking TG2 display an impaired mitochondrial function associated to an increase in their aerobic glycolysis and become sensitive to the glycolytic inhibitor 2-deoxy-D-glucose (2-DG) [32]. Based on these findings we decided to focus our attention on PKM2. To understand the biological role of the TG2/PKM2 interaction, we first performed the RNA interference for PKM2 in 2fTGH cells and analysed the expression of TG2. PKM2 knockdown was successful as indicated by the western blotting analysis (Figure 3A and 3B). Considering that the absence of PKM2 causes alterations in glycolysis pathway [37], we asked how PKM2 down-regulation affects the overall cell metabolism. We therefore measured the lactate concentration, as an indicator of the glycolytic flux, in 2fTGH cell lines in the presence and absence of PKM2. Lack of PKM2 resulted in significantly less glycolytic activity that in turn caused a decrease in the acidification of the culture medium (Figure S3). In keeping with these findings we detected a marked reduction in the level of HIF1, a key regulator of glycolysis (Figure 3A and 3B) [41]. In order to verify whether the PKM2 ablation and the consequent glycolysis inhibition had any effect on TG2, we monitored the enzyme's transamidating activity upon autophagy induction by using rapamycin, a well-known lipophilic macrolide antibiotic which is widely used to induce autophagy by inactivating mTOR, and 2-DG a glycolysis inhibitor. To this aim, we used BAP in corporation assay to detect the intracellular TG2's transamidating activity. Intracellular TG2 is known to be inactive under steady state conditions and activated upon stressful stimuli, which lead to the accumulation of free Ca^2+^ inside the cytoplasm [7, 8]. Figure 3C clearly indicates a net decrease of the intracellular enzyme's transamidating activity after 4 hours of rapamycin treatment in the absence of PKM2 with no effect observed by the treatment with 2-DG. To confirm the effect of the PKM2 ablation on TG2's transamidating activity we monitored the crosslinking activity in cells starved for 24 and 48h (Figure 3D). Taken together these results demonstrate the marked inhibitory effect displayed by PKM2 ablation on the increase of the TG2's transamidating activity (Figure 3E) observed upon autophagy induction with both rapamycin and starvation (Figure 3C and 3D).

In order to have an insight on the molecular mechanisms by which PKM2 regulates the TG2's transamidating activity, we examined the TG2 phosphorylation upon PKM2 ablation (Figure 3F). To this aim we immunoprecipitated TG2 in 2fTGH cells in which PKM2 was silenced by siRNA and the level of tyrosine-phosphorylated enzyme was detected by western blot analysis using a phospho-tyrosine antibody. The results reported in Figure 3F clearly indicated that, upon silencing of PKM2, there is a marked increase in the phospho-tyrosine staining of TG2 compared to the control, thus suggesting that the decreased TG2's transamidating activity correlates with an increased in its tyrosine post-translational modification.

### Downregulation of PKM2 leads to autophagy impairment

**Figure 4:**
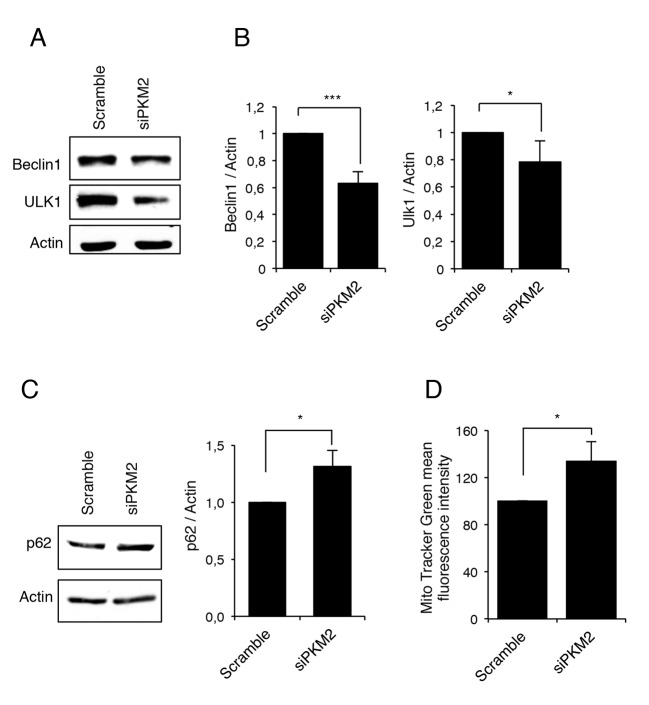
PKM2 inhibition leads to an autophagy impairment **A**. Immunoblotting analysis of Beclin1 and ULK1 in 2fTGH cells after RNA interference for PKM2. Actin was used as a loading control. **B**. Densitometric analysis of blots (quantification of Beclin1 and ULK1 bands normalized to Actin levels). Data are expressed as mean ± SD, n = 4 independent experiments. Statistical significance refers to the respective control (*p<0.05 and ***p<0.0001). **C**. Immunoblotting analysis of p62 in 2fTGH cell line in the presence and absence of PKM2 and densitometric analysis of blots (quantification of p62 bands normalized to Actin levels). Data are expressed as mean ± SD, n = 4 independent experiments. Statistical significance refers to the respective control (*p<0.05). Actin was used as a loading control. **D**. Mitochondrial mass was evaluated in 2fTGH cell lines in the presence and absence of PKM2 by MTG staining and quantified by FACS. Quantification of MTG mean fluorescence intensity is referred as percentage of a control value. Data are expressed as mean ± SD, n = 3 independent experiments. Statistical significance refers to the respective control (*p<0.05).

Previous studies have shown that the TG2 transamidating activity is involved in the last stages of autophagy [28-29]. On the other hand, we and others have demonstrated that PKM2 is involved in the regulation of HIF1 expression [42-43] and according to this, a decreased glycolysis has been linked to a decreased expression of HIF1 and autophagy inhibition [41]. Considering the above reported effect of PKM2 on TG2, we decided to verify whether PKM2 knockdown could also affect autophagy. Therefore, we first monitored autophagy after PKM2 inhibition by analysing the expression levels of Beclin1 and ULK1, two key proteins involved in the early stages of autophagosome formation [44]. Figure 4A and 4B clearly show that in the absence of PKM2 there is a significant decrease of Beclin1 and ULK1 suggesting impairment in the early step of autophagosome formation. To verify this hypothesis, we decided to better characterize the autophagic process in the presence and absence of PKM2 by monitoring p62 (Figure 4C), an autophagy cargo protein that is itself degraded by autophagy and functions to deliver ubiquitinated proteins to the phagophore by binding directly to LC3 and thus enabling their degradation in the lysosome [45-47]. Interestingly, the inhibition of PKM2 resulted in significant accumulation of p62 thus confirming a possible inhibition in the autophagic process (Figure 4C) [45].

Autophagy is the major mechanism to eliminate damaged organelles and we have recently demonstrated that TG2, by its transamidating activity, is also involved in the removal of damaged mitochondria by this process [32]. Considering the above reported autophagy impairment associated with the absence of PKM2, we decided to verify whether mitophagy might be affected as well. For this aim, we treated 2fTGH cells expressing different levels of PKM2 with the mitochondrial uncoupler carbonyl cyanide m-chlorophenyl hydrazine (CCCP) for 18 h and measured their relative mitochondrial mass by Mito Tracker Green (MTG) staining and flow cytometry analysis. As shown in Figure 4D, the cells with down-regulated PKM2 exhibit a significant increase in mitochondrial mass compared to the controls, confirming that the PKM2-dependent impairment of autophagy also leads to a defective clearance of mitochondria.

### Loss of PKM2 leads to a defect in autophagic flux

**Figure 5:**
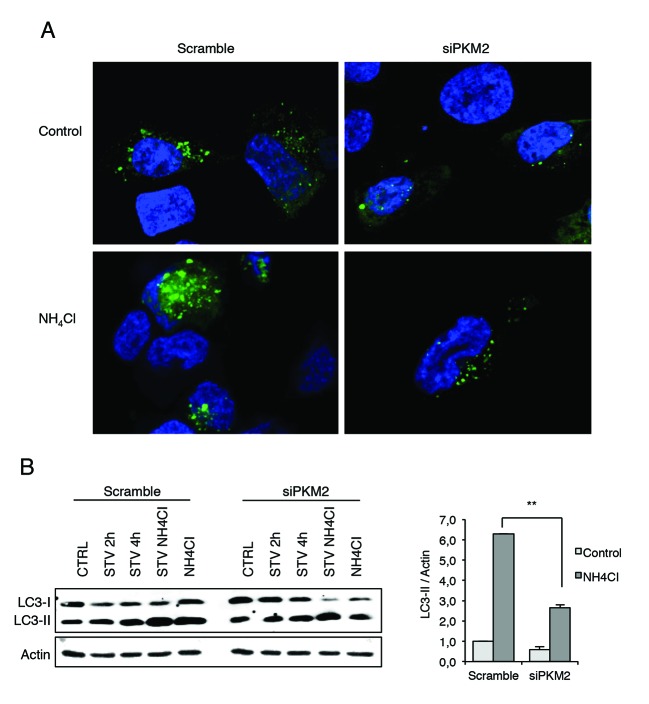
Immunofluorescence analysis of autophagy in the presence and absence of PKM2 **A**. Analysis of autophagic flux in GFP-LC3 overexpressing 2fTGH cells and in the presence of NH_4_Cl, which inhibits the activation of the lysosomal enzymes, hence blocking the degradation process of LC3 II isoform. Green dots represent autophagosomes, nuclei were stained with Hoechst 33342 (blue). **B**. Immunoblotting analysis of LC3 in 2fTGH cells starved for 2 h and 4 h in EBSS or treated with 20 mM NH_4_CI for 4 h after RNA interference for PKM2. Proteins were analysed by immunoblotting using anti-LC3 (left panel). Densitometric analysis of blots (quantification LC3 II bands normalized to Actin levels). Data are expressed as mean ± SD, n = 4 independent experiments. Statistical significance refers to the respective control (**p<0.001) (right panel).

In order to get an insight into PKM2's role in the autophagic process, we decided to investigate the effect of its ablation in 2fTGH cells overexpressing GFP-LC3, which is widely used as a marker for autophagic vesicles [47]. To detect the autophagic flux we also analysed these cells in the presence and in the absence of NH_4_Cl. As clearly shown in Figure 5A, in the absence of PKM2, GFP-LC3 dots decreased compared to the control samples. This finding is further confirmed by blocking the autophagy flux by NH_4_Cl that leads to an increased number of dots only in the control cells, further indicating a defect in autophagy in the absence of PKM2. These results were confirmed by the western blot analysis of the LC3II levels observed upon ablation of PKM2 by siRNA in cells treated with NH_4_Cl. In fact, the results reported in Figure 5B show that upon blockade of the lysosomal activity by NH_4_Cl, the cells lacking PKM2 display a significative reduction in the autophagic flux as indicated by the decreased accumulation of LC3II upon NH_4_Cl treatment.

## DISCUSSION

The results of this study confirmed the complexity of the biological role played by TG2. The analysis of the enzyme's proteome has revealed that TG2 interacts with various categories of proteins of which the most represented is a well-defined group of chaperones involved in proteostasis. The composition both in terms of number of proteins detected and the categories represented is largely reduced upon induction of autophagy. Interestingly, the comparative analysis of the two interactomes also reflects that, upon induction of autophagy, TG2 is in its transamidating-prone open conformation while under steady state conditions the enzyme is inactive and is present in its close conformation [8].

It is also important to mention that previous studies reported some of the proteins we detected in the interactome as TG2 substrates or interactors (tubulin, actin, ANT and gyceraldehyde-3-phosphate dehydrogenase) thus validating that the TG2 partners reported in this study are *bona fide* enzyme's specific interacting proteins [14, 48-49]. Interestingly, previous studies have revealed that TG2 is a fundamental component for proper maturation of autophagosomes and that TG2 ablation results in mitophagy impairment and a shift to high levels of glycolysis indicating an alteration in the mitochondrial metabolism [29, 32]. Our interactome analysis demonstrated that TG2 interacts with PKM2, which is a key rate limiting enzyme controlling the cellular glycolytic activity [50].

Regulation of PKM2 activity by tyrosine kinase signalling is important for metabolic changes that support proliferative metabolism and tumor growth in several contexts [50]. In line with this finding, previous studies have shown that TG2 interacts with Fructose-1,6-bisphosphate aldolase, glyceraldehyde-3-phosphate dehydrogenase, alpha-ketoglutarate dehydrogenase, phospho-glycerate dehydrogenase and fatty acid synthase which are involved in energy metabolism [51-52]. All these findings suggest a role for TG2 in the regulation of energy homeostasis. Interestingly, our data show that the PKM2 inhibition causes a blockage in autophagy. It has been well documented that tumor cells activate autophagy in response to a variety of stresses [53-54]. Indeed, inhibition of PKM2 leads to a significant reduction in the expression of early regulators of autophagy such as and ULK1 and Beclin1 associated to a decrease in LC3II processing and consequently an impairment of the autophagic flux as also demonstrated by accumulation of p62. In keeping with these findings PKM2 inhibition also leads to a significant reduction in the expression of HIF1, a well-known positive regulator of autophagy [41]. Interestingly, it has been shown that dimeric PKM2 localizes to the nucleus, where by acting as a protein kinase activates STAT3 [55-56]. Nuclear STAT3 modulates autophagy via the transcriptional regulation of several autophagy-related genes, such as BCL2, BECN1, PIK3C3, HIF1, BNIP3, and various microRNAs, including the MIR17HG that regulates both ULK1 and Beclin1 [57-58]. These findings strongly support our data showing that the ablation of PKM2 leads to autophagy impairment characterized by Beclin1, ULK1, HIF1 protein downregulation. Interestingly, the down-regulation of PKM2 leads to a marked decrease of TG2 transamidating activity indicating that PKM2 is able to stimulate TG2 cross-linking activity that is required for the proper maturation of autophagosomes [29, 32]. Thus, under steady state conditions PKM2 might positively regulate autophagy by modulating, either directly or indirectly, the TG2's transamidating activity. In keeping with this assumption, it is interesting to note that, in the absence of PKM2, we observed an increased TG2's phosphorylation on tyrosine residues. Thus this indicates that PKM2 negatively regulates a tyrosine kinase(s) that are able to post-translationally modify TG2. Based on these results it is possible to hypothesize that the negative regulation carried out by TG2 on glycolysis involves its interaction with PKM2. In conclusion, this study has highlighted for the first time an important homeostatic control linking autophagy to glycolysis, which is dependent by the relative expression levels of PKM2 and its interplay with TG2. Future studies should further define the exact molecular events regulating this important cellular metabolic switch which is a typical hallmark of cancer cells. It is important to note that the modulation of autophagy has been shown to have high therapeutical impact in tumor treatment [59-62].

## MATERIALS AND METHODS

### Antibodies

The following antibodies were used: anti-TG2 (Thermo Scientific, CUB7402), anti-PKM2 (Cell Signalling, D78A4), anti-EEF1A1 (Milipore, 05-235), anti-Hsc70 (Abcam, 51052), HSPA1A (Santa Cruz, 7947), anti-Biotin (Abcam, 1227), anti-p62/SQSTM1 (mbl, PM045), anti-LC3 (Novus Biologicals, NB100-2331), anti-GAPDH (Sigma, G9545), anti-Actin (Sigma, 2066), anti-Beclin1 (Santa Cruz, 10086), anti-HIF1-beta (Novus Biologicals, NB100-124), anti-ULK1(Santa Cruz, 33182), anti-phospho-tyrosine (Cell Signaling, 9411) and HRP-conjugated secondary antibodies (Bio-Rad Laboratories).

### Cell culture, transfection and drug treatments

2fTGH (human fibrosarcoma), Flag-HA-TG2 2fTGH, GFP-LC3 2fTGH and RFP-GFP-LC3 2fTGH cell lines were cultured in Dulbecco's modified Eagle's medium (Lonza) supplemented with 10% fetal bovine serum, 2 mM L-glutamine, 100 μg/ml streptomycin and 100 units/ml penicillin at 37°C. For generation of the TG2 stably expressing cells (Flag-HA-TG2 2fTGH), 2fTGH cells were transfected with pLPCX-TG2-HA-Flag using CaCl_2_ method. Autophagy was induced by using 1 μM rapamycin (Rapam, Sigma-Aldrich) or 10 mM 2-deoxy-D-glucose (2-DG, Sigma-Aldrich). For amino acids starvation (Starv), cells were washed twice in the amino-acid-free medium Earle's Balanced Salt Solution (EBSS) (Sigma-Aldrich) and incubated in the same medium for the indicated periods. In order to inhibit autophagy, cells were incubated in full medium with 20 mM ammonium chloride (NH_4_Cl, Sigma-Aldrich) for the indicated period. For proteasome inhibition, cells were incubated with 5 μM MG132 (Z-Leu-Leu-Leu-al; Sigma Aldrich) for 4 h. To induce mitochondrial damage, cells were incubated in full medium with 15 μM carbonyl cyanide m-chrolorophenyl hydrazine (CCCP, Sigma-Aldrich). TG2 transamidating activity was inhibited by incubating the cells with 40 μM Z-DON (Zedira) for 24 hours, concomitantly with starvation.

### Cloning of TGase 2 gene with HA and Flag tag

HA tagged TG2 was obtained by polymerase chain reaction (PCR) amplification using pLPCX-TG2 vector. The construct was cloned into the *XhoI* and *EcoRI* sites of the pLPCX-TG2 vector. Second, pLPCX-TG2-HA plasmid was used as a template to produce dual tag. The construct was inserted into the *NotI* and *Xhol* sites of the template. The plasmid containing full length human TG2 tagged with HA and Flag was transformed and sequenced to verify the amplification. Primers used for plasmid construction of TG2 HA-Flag are shown in Figure S1D.

### Retrovirus generation and infection

5 mg of an expression plasmid (pLPCX TG2-HA-Flag) was co-transfected with 15 μg of the retroviral vector into HEK293 gp/bsr using the calcium phosphate method. After 48 h, the supernatant containing the retroviral particles was collected and supplemented with polybrene (4 μg/ml). 2fTGH cell lines were infected with retroviral-containing supernatant for 6–8 h.

### Tandem Affinity Purification (TAP)

Cell lysates were prepared in lysis buffer (10 mM Tris, 50 mM NaCl, 10% glycerol, 0.5% NP40) and protease inhibitors. The lysates were pre-cleared by using IgG beads for 45 min and incubated with 200 μl Flag agarose beads for 2 h and washed extensively. To elute the bound proteins, the supernatant was incubated with Flag peptide for 30 min at 4°C and centrifuged again. The eluted complex was reincubated with 100 μl HA agarose beads for 2 h and washed 5 times with 1 ml Tris/NP40. The final protein complex was eluted with 100 μl Glycine (100 mM, pH: 2.4). 10 μl of Tris-Cl (1 M, pH: 8.0) was immediately added to neutralize the effect of glycine. The complex containing TG2 and its interacting proteins were separated by HPLC and identified in mass spectrometry.

### Gel sypro

The gel was (1) fixed in 10% methanol: 7% ethanoic acid for 30 min with continuous rocking; (2) incubated over-night with Sypro Stain (RubyTM Gel Stain 1X, BioRad); (3) washed in Fixer solution and in deionized water for 30 min and then visualised at Typhoon scanner.

### Liquid sample processing for HPLC analysis

The purified complexes of TG2 with its interacting partners obtained from TAP were boiled for 5 min at 100°C to denature the proteins and expose the hidden sites to trypsin action. The disulphide bonds were reduced with 10 mM dithiothreitol at 56°C for 30 min, and alkylated with 55 mM iodoacetamide at room temperature for 20 min. The samples were then precipitated in 80% ethanol and incubated over-night at −80°C.

The following day, the samples were centrifuged at 4000 g at 10°C for 40 min. The protein pellet was resuspended in a solution of 50 mM ammonium bicarbonate at room temperature. The samples were digested by trypsin (200 ng) at 37°C for 12 h, purified through the filter of a Zip-Tip (C-18 Resin, Millipore) and eluted from the Zip-Tip with 80% acetonitrile and 0.1% TFA. To eliminate the excess of acetonitrile, speedvac was used for 3 min and resuspended in 7 μl of 2.5% acetonitrile and 0.1% TFA. The digested peptides were separated through the HPLC by their different hydrophobicity and spotted on a MALDI plate.

### MALDI-mass spectrometry

MALDI-mass spectrometry (MS) and MALDI-MS/MS were performed on an Applied Biosystems 4700 Proteomics Analyzer with TOF/TOF ion optics (Applied Biosystems, Foster City, CA, USA). Spectra were calibrated externally using five peaks of standard (ABI4700 Calibration Mixture; Applied Biosystems) or internally using porcine trypsin autolysis peptide peaks. In addition to peptide mass finger spectra, the five most abundant pre-cursor ions masses having a signal-to-noise ratio >50 were chosen for MS/MS fragmentation. The interpretation of both the MS and MS/MS data were carried out with the GPS Explorer software (version 3.6; Applied Biosystems). Peaks were extracted from raw spectra by the GPS Explorer software. A combined MS peptide fingerprint and MS/MS peptide sequencing search was performed against the NCBI non-redundant database without taxon restriction using the MASCOT search algorithm. MS/MS peptide spectra with a minimum ion score confidence interval ≥95% were accepted; this was equivalent to a median ion score cut-off of approximately 35 in the data set. Protein identifications were accepted with a statistically significant MASCOT protein search score ≥76 that corresponded to an error probability of P<0.05 in our data set.

### Immunoblotting

Cells were collected in a lysis buffer containing 20 mM Tris-HCl pH 7.4, 150 mM NaCl and 1% Triton X-100 with protease inhibitor cocktail (Roche). Proteins were quantified by standard Bradford staining and separated using SDS polyacrylamide gels. Nitrocellulose membranes were used for transfer and blots were blocked in 5% non-fat dry milk for 1 h at room temperature and then incubated overnight with the primary antibodies. The membranes were incubated with HRP conjugated secondary antibody for 1 h at room temperature and the signal was detected by Immun-Star WesternC Kit (Bio-Rad Laboratories).

### Co-immunoprecipitation assays

The cells were harvested with a lysis buffer (10 mM Tris-HCl pH 8.0, 150 mM NaCl, 10 % Glycerol, 0.5% NP40 and proteases inhibitors), incubated at 4°C for 30 min and centrifuged at 15000 g for 10 min at 4°C. The lysates were incubated with IgG beads for 45 min for pre-clearing. The supernatants were incubated with anti-TG2 antibody overnight in rotation. The following day, the complex solution were incubated with IgG beads for 45 min and then centrifuged. The precipitate was eluted with 35 μl of sample buffer 2X including β-mercaptaethanol, boiled at 95°C and used for western blot. Immunoblot analysis was performed with indicated antibodies.

### RNA interference

siRNA oligos corresponding to the human PKM2 were from Gene Script. 200000 cells per well were transfected with 10 nM final concentration of siRNA by Lipofectamine 2000 (Invitrogen) according to the manufacturer's supply. After 72 h of transfection, cells were collected in lysis buffer. The RNA interference was checked by western blot.

### *In situ* TG assay

In situ TG activity was measured by incorporation of 5-(biotinamido)pentylamines (BAP) into protein substrates. 2 mM BAP (Soltec Ventures, B110) were directly added into the medium together with the indicated treatments. In the presence of TG2 transamidating activity, BAP is incorporated into the substrates. To measure this activity, cells were lysed as above described and proteins were resolved by SDS-polyacrylamide gel. The blots were performed as above mentioned and anti-Biotin antibody was used as a primary antibody.

### Confocal microscopy

GFP-LC3 2fTGH, grown on coverslips, were fixed in 4% paraformaldehyde for 10 min and washed with PBS three times. Nuclei were stained with 10 mg/ml Hoechst 33342 for 10 min. The coverslips were mounted in antifade (Fluorogel with Dapco, Eletron Microscopy Sciences) and examined under a confocal laser scanner microscope (Olympus FV 1000).

### Detecting of lactate levels

Colorimetric Lactate Assay Kit (Abcam) was used to measure lactate. The assay was performed in 96-well plate, according to manufacturer's instructions. The reaction was incubated for 30 min at RT (protected from light) and measured using a 96-well plate reader at absorbance 570 nm.

### Analysis of mitochondrial mass

Mitochondrial mass was measured by Mito Tracker Green (MTG, Molecular Probes) staining. Cells were trypsinized, washed and resuspended in PBS with 100 nM MTG for 30 min at 37°C. Fluorescence was measured by FACScan flow cytometer (BD Biosciences) using the CellQuest software (BD). In each analysis, 10000 events were recorded.

### Statistical analysis

Densitometry measurements were used for statistical analysis using Graph Pad. Statistical significance was determined using the Student's t test; p<0.05 was considered significant.

## SUPPLEMENTARY MATERIAL FIGURES



## Abbreviations

2-DG: 2-deoxy-D-glucose
BAP: 5-(biotinamido)pentylamine
2fTGH: human fibroblasts
MTG: Mito Tracker Green
TG2: Transglutaminase 2
PKM2: pyruvate kinase M2
