# Supplementary material for: The transglutaminase type 2 and pyruvate kinase isoenzyme M2 interplay in autophagy regulation
Source: Oncotarget. 2015 Dec 24;6(42):44941–54. doi: 10.18632/oncotarget.6759 (PMC4792602; doi:10.18632/oncotarget.6759)
Supplement: Supplementary file 1 [file oncotarget-06-44941-s001.pdf]

The transglutaminase type 2 and pyruvate kinase isoenzyme M2 interplay in autophagy regulation

Supplementary Material

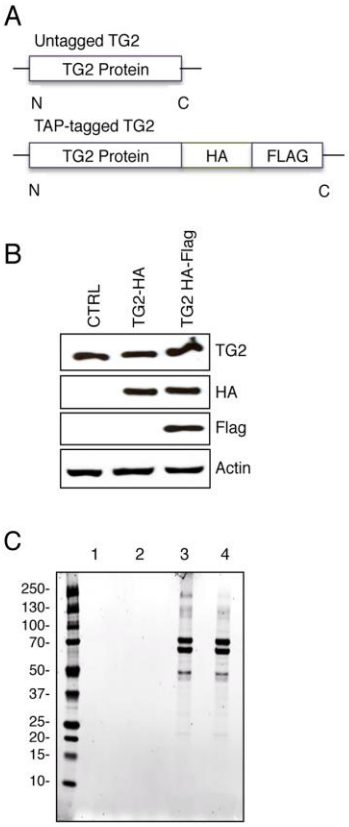

D

Primers	Sequence
TG2-HA-F	CCG CTC GAG ATG GCC GAG GAG CTG GTC TTA GAG AGG TGT G
TG2-HA-R	GCGA ATT CTA AGC GTA ATC TGG AAC ATC GTA TGG GTA GGC GGG GCC AAT GAT GAC ATT CCG G
TG2-Flag-R	TAG AGC GGC CGC TAT TTA TCG TCA TCG TCT TTG TAG TCA GCA GCG TAA TCT GGA ACA TCG TAT GGG TA

Figure S1. The tandem affinity purification (TAP) procedure. (A) Schematic representation of untagged and TAP-tagged TG2. (B) Immunoblotting analysis of TG2, HA and Flag in Flag-HA-TG2 overexpressing 2fTGH cells. (C) Extracts of 2fTGH cells transfected with untagged or TAP-tagged TG2 were subjected to TAP. For autophagy induction, cells were washed two times

in EBSS and incubated in EBSS for 2 h. Final TAP elutes were analyzed by Sypro staining. 1: 2fTGH cells expressing untagged TG2 under fed conditions; 2: 2fTGH cells expressing untagged TG2 subjected to starvation; 3: 2fTGH cells expressing TAP-tagged TG2 under fed conditions; 4: 2fTGH cells expressing TAP-tagged TG2 subjected to starvation. (D) Primer sequences used for TG2 HA-Flag plasmid construction.

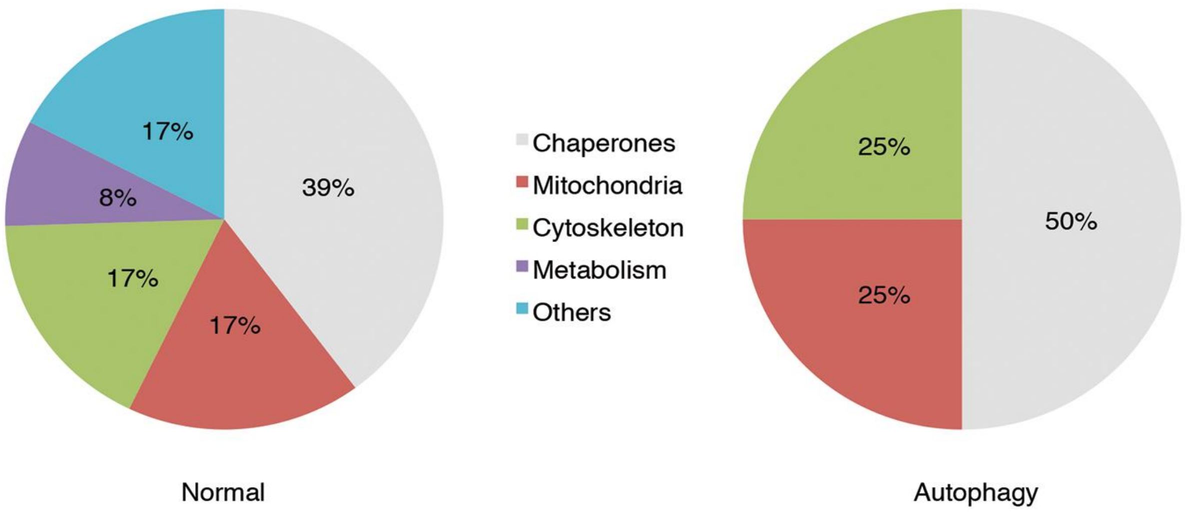

Figure S2. **Overview of the proteomic analysis results showing the categories of TG2 interacting proteins under normal and autophagic conditions.**

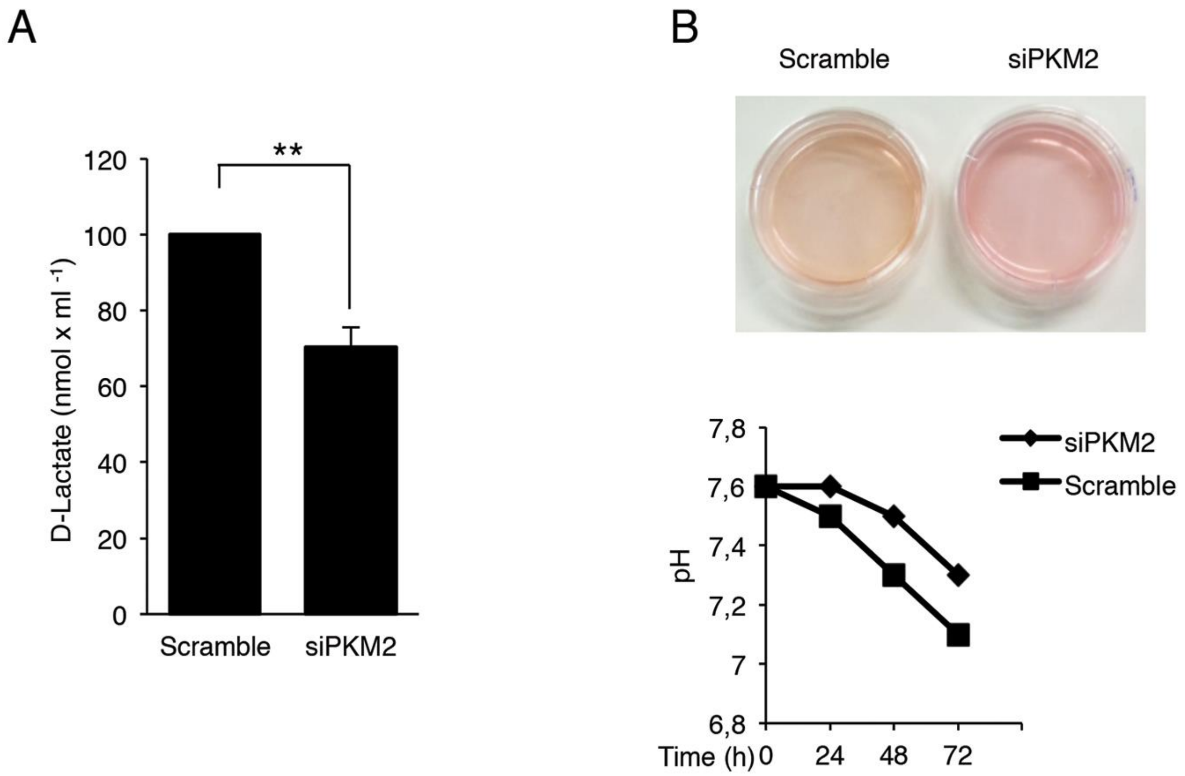

Figure S3. Ablation of PKM2 leads to decrease in glycolysis in **2fTGH cells. (A) Tissue culture medium was used to measure L-lactate production as described in Material and Methods.** Data are expressed as mean ± SD, n = 3 independent experiments. Statistical significance refers to the respective control (**p<0.001). (B, upper panel) **Representative experiment showing cell medium acidification after 3 days of culture in 2fTGH cells in the presence and absence of PKM2. (B, lower panel) pH determination at different time points in 2fTGH cell lines after RNA interference of PKM2.**
